# Improved atomic force microscopy cantilever performance by partial reflective coating

**DOI:** 10.3762/bjnano.6.150

**Published:** 2015-07-03

**Authors:** Zeno Schumacher, Yoichi Miyahara, Laure Aeschimann, Peter Grütter

**Affiliations:** 1 Department of Physics, McGill University, Montreal, Quebec, H3A 2T8, Canada; 2 NanoWorld AG, Neuchâtel, 2002, Switzerland

**Keywords:** cantilever, force noise, partial coating, *Q*-factor

## Abstract

Optical beam deflection systems are widely used in cantilever based atomic force microscopy (AFM). Most commercial cantilevers have a reflective metal coating on the detector side to increase the reflectivity in order to achieve a high signal on the photodiode. Although the reflective coating is usually much thinner than the cantilever, it can still significantly contribute to the damping of the cantilever, leading to a lower mechanical quality factor (*Q*-factor). In dynamic mode operation in high vacuum, a cantilever with a high *Q*-factor is desired in order to achieve a lower minimal detectable force. The reflective coating can also increase the low-frequency force noise. In contact mode and force spectroscopy, a cantilever with minimal low-frequency force noise is desirable. We present a study on cantilevers with a partial reflective coating on the detector side. For this study, soft (≈0.01 N/m) and stiff (≈28 N/m) rectangular cantilevers were used with a custom partial coating at the tip end of the cantilever. The *Q*-factor, the detection and the force noise of fully coated, partially coated and uncoated cantilevers are compared and force distance curves are shown. Our results show an improvement in low-frequency force noise and increased *Q*-factor for the partially coated cantilevers compared to fully coated ones while maintaining the same reflectivity, therefore making it possible to combine the best of both worlds.

## Introduction

For cantilever based beam deflection atomic force microscope (AFM) systems, a large variety of commercial cantilevers exist. For each measurement mode, e.g., tapping, contact, non-contact, etc. optimized cantilevers are offered. These cantilevers differ in parameters like dimension, spring constant, resonance frequency and tip size. Most cantilever models are available in two versions, an uncoated version and a version with a reflective metal coating. The reflective coating is added to enhance the poor intrinsic reflectivity of silicon, the material most cantilevers are made of. On average adding a reflective coating increases the intensity of the reflected beam by 2.5 times, hence resulting in higher signals on the photodiode.

In frequency modulated (FM) AFM, the mechanical quality (*Q*-)factor of the cantilever plays an important role, since the measurable minimal force gradient in FM-AFM is [1]:

[1]
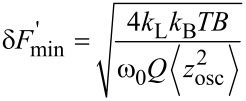


where *Q* is the mechanical *Q*-factor of the cantilever, *k*_L_ the force constant of the cantilever, *T* the temperature, *k*_B_ the Boltzman constant, *B* the measurement bandwidth, ω_0_ the resonance frequency of the cantilever and 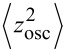 the root-mean-square amplitude of cantilever oscillation. Since the minimal detectable force is inversely proportional to the *Q*-factor, high *Q*’s are desired to achieve a lower minimal detectable force gradient. By using a cantilever in an ultra high vacuum environment (UHV), the *Q*-factor is drastically increased due to the absence of damping by air atmosphere and is limited by the intrinsic properties of the cantilever.

It is known that adding a metal layer to a cantilever can degrade the *Q*-factor of the cantilever. A reduction in *Q*-factor due to a metallic coating of >100 nm thick film [2] and of thinner films [3] have been reported.

Another undesirable effect caused by a metallic coating is the increased low-frequency noise which often exhibits an 1/*f* behavior. Labuda et al. recently published a study on how to reduce the 1/*f* noise of coated cantilevers by patterning the metal coating with a Fresnel lens like pattern [4]. Bull et al. reported the reduction of the cantilever noise in liquid by a partial metallic coating on commercially available short cantilevers [5]. These changes in the cantilever performance can be described by the additional viscoelastic damping and increased susceptibility to temperature fluctuations due to the added metal layer causing a bimetallic effect. Paoline et al. presented a model that uses a complex spring constant in combination with Sader’s model of hydrodynamic damping to describe the 1/*f* noise behaviour of coated cantilever [6].

Since the sole purpose of the reflective coating is to increase the intensity of the reflected light, it is only needed at the position of the incident laser beam, i.e., at the tip end of the cantilever. Waggoner et al. presented a study on the effect of a circular gold pad at different positions along a cantilever showing a reduction in *Q*-factor for pads placed at the base of the cantilever [7]. Sosale et al. reported a study on partially metalized cantilevers and the resulting *Q*-factor, finding an optimal coating length of 20% at the tip end and high damping due to coating at the base [8–9]. However, they used cantilevers with dimensions of 22.6 to 24.1 mm in length and 73 to 93 μm in thickness with a coating thickness of 110 nm which are mounted on a custom-made holder for minimizing clamping losses. Although, these cantilevers do work well as a model system, they do not represent the dimensions of commonly used commercial cantilevers for AFM.

It is widely believed that a source of the variability of the *Q*-factor of commercial cantilevers is a bad coupling between the piezo and the cantilever and the resulting clamping losses [10]. We will present a study of the effect of the reflective coating on the *Q*-factor and the noise of commercially available cantilevers and how these influence the performance in the different AFM operation modes. We will provide evidence that a small change in coating thickness can influence the *Q*-factor significantly.

## Experimental

We measured the dependencies of low-frequency noise and *Q*-factor on partial metal coating coverage. As previously mentioned, different AFM modes require different cantilevers. Two types of cantilevers were chosen for this study. First, a soft (≈0.01 N/m) cantilever mainly used for contact mode and force–distance measurements, where a low spring constant and low 1/*f* noise are the most important parameters. Second, a stiff (≈29 N/m) cantilever typically used in high resolution UHV AFM applications, where the focus is on the *Q*-factor, was used. Cantilever specifications are summarized in Table 1. The partial reflective coating was realized by a shadow masking technique with thermal evaporation. The length of the partial coating, as well as the length of the cantilevers were measured with a calibrated optical microscope, with an estimated error of ±1 μm.

**Table 1:** Specification of the two types of cantilevers used for this study.

Cantilever name	Soft	NCLR

Spring constant	≈0.01 N/m	≈29 N/m
Length	140 μm	225 μm
Width	34 μm	38 μm
Thickness	340 nm	7 μm
Coating thickness	2 nm Cr & 60 nm Au	30 nm Al
Ratio coating/substrate thickness (*h*_f_/*h*_s_)	3/17	3/700
Tested coating percentages	15, 19, 21, 26, 32, 55, 60, 100	0, 20, 24, 27, 32, 41, 44, 48, 100

All measurements were performed with a variable-environment compatible commercial AFM (JEOL JSPM-5400) under high vacuum conditions (<5 × 10^−5^ mbar) or in air atmosphere. The standard laser diode was replaced with a fiber-pigtailed, temperature stabilized and radio frequency modulated laser diode to reduce the mode-hopping noise of the laser beam [11]. The standard cantilever holder with a metal wire across the chip that clamps the cantilever was used for all measurements.

The cantilever deflection noise density spectra were obtained by fast Fourier transform of the digitized photo diode signal. An 8th order Buttherworth low-pass filter with an appropriate cut-off frequency was used as an anti-aliasing filter. The resulting thermal vibration peak was fitted with a Lorentzian to extract its full width at half maximum from which the *Q*-factor was calculated. The spring constant of the cantilevers and optical-lever sensitivities were measured by fitting the thermal vibration peak of the fundamental flexural mode acquired in air. The obtained optical-lever sensitivities are used to convert the noise density spectra to be expressed in fm/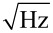. More detail of the procedure is found in [12].

The *Q*-factor was also obtained by exciting the cantilever oscillation with a piezoelectric actuator and measuring the resulting amplitude and phase with varying frequency with a digital lock-in amplifier (HF2LI, Zurich Instrument). The *Q*-factor was calculated from the measured phase versus frequency curves using 
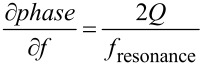
.

## Results and Discussion

The sum signal measured on the photodiode was the same for the partially coated cantilever and the fully coated cantilever under constant laser power. The uncoated cantilever shows a 2 and 3 times lower reflectivity for the NCLR and soft type respectively. In the following paragraph we will highlight the advantage of a partial reflective coating on NCLR and Soft cantilevers for FM-AFM and static AFM operation, respectively.

### Advantages for FM-AFM: recovering intrinsic *Q*-factor values

**Figure 1:**
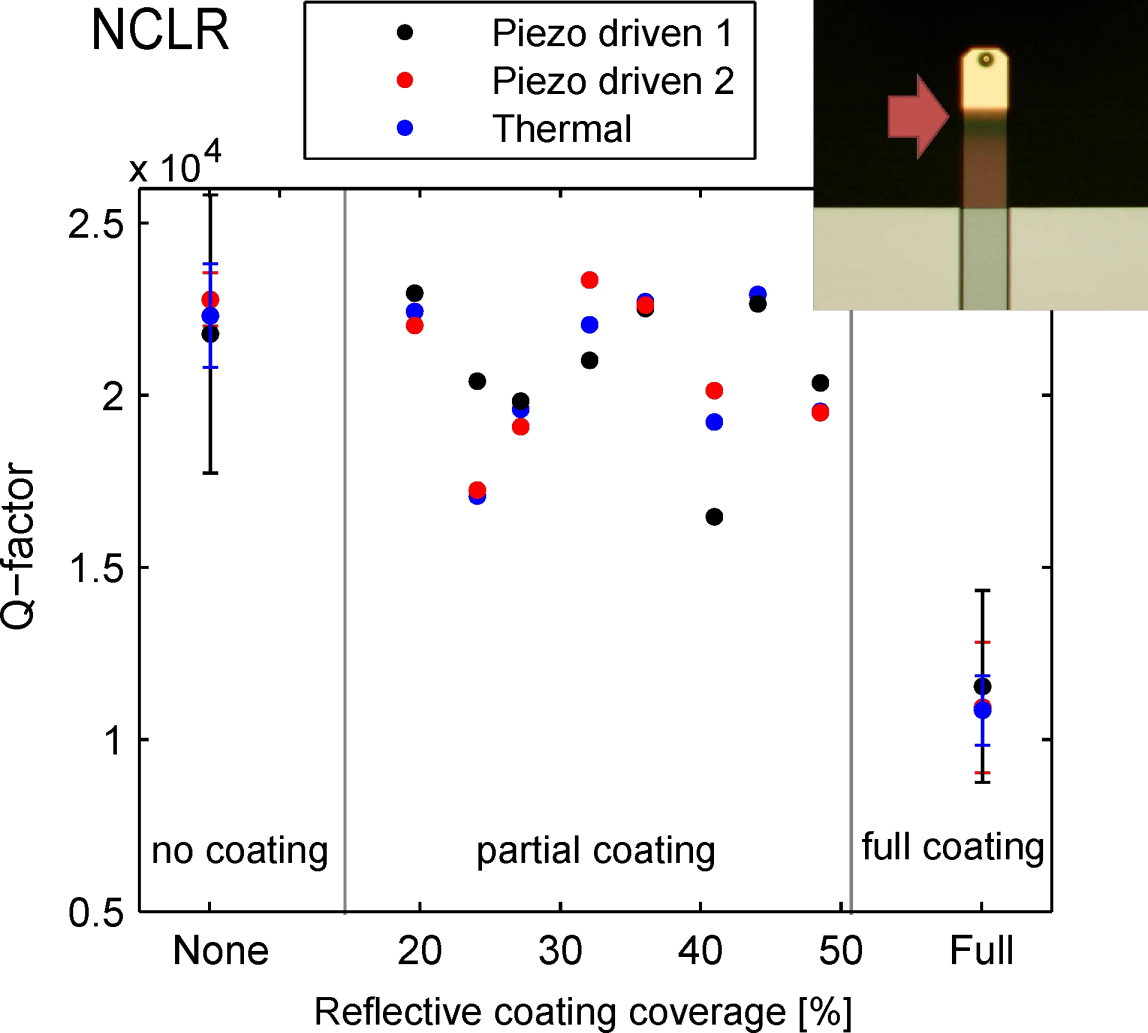
*Q*-factor of NCLR cantilever with different coating coverage percentages. A 30 nm Al coating was added on the 7 μm thick cantilever. 20% coating coverage show the same *Q*-factor as uncoated cantilever. The errorbars show standard deviation of the mean of at least 3 different cantilevers.

Figure 1 shows the *Q*-values for the NCLR cantilever with different coating coverages measured in high vacuum. For each of the uncoated and fully coated cantilevers, the average of at least 3 different cantilevers is plotted. As previously mentioned, the minimal detectable force in FM-AFM can be reduced by increasing the *Q*-factor. Adding a full reflective coating to the NCLR cantilevers reduces the *Q*-factor by half compared to uncoated cantilevers. However, by minimizing the coating to 20% of the cantilever length the same *Q*-factor as that of the uncoated cantilever can be achieved.

A thermal vibration measurement (blue) is compared to a piezo driven measurement recorded during the same experiment (Piezo driven 2) and a piezo driven experiment recorded after re-mounting of the cantilever (Piezo driven 1). The *Q*-factor varies slightly between the thermal and the piezo driven measurement performed with the same clamping. This difference is attributed to multiple possible sources. The thermal vibration measurement is more susceptible to temperature drift as it requires longer acquisition time for measuring the cantilevers with higher *Q*-factors. The fitting can also contribute to a difference in the measured values due to the high *Q*-factor. The variation between the two piezo-driven measurements stems from the difference in mounting and therefore possible different clamping losses [10].

In addition, the soft cantilevers show even more pronounced effects under vacuum due to the different coating and cantilever thickness (see Supporting Information File 1, Figure S1 for data).

### Advantages for static AFM: reduced low-frequency noise

**Figure 2:**
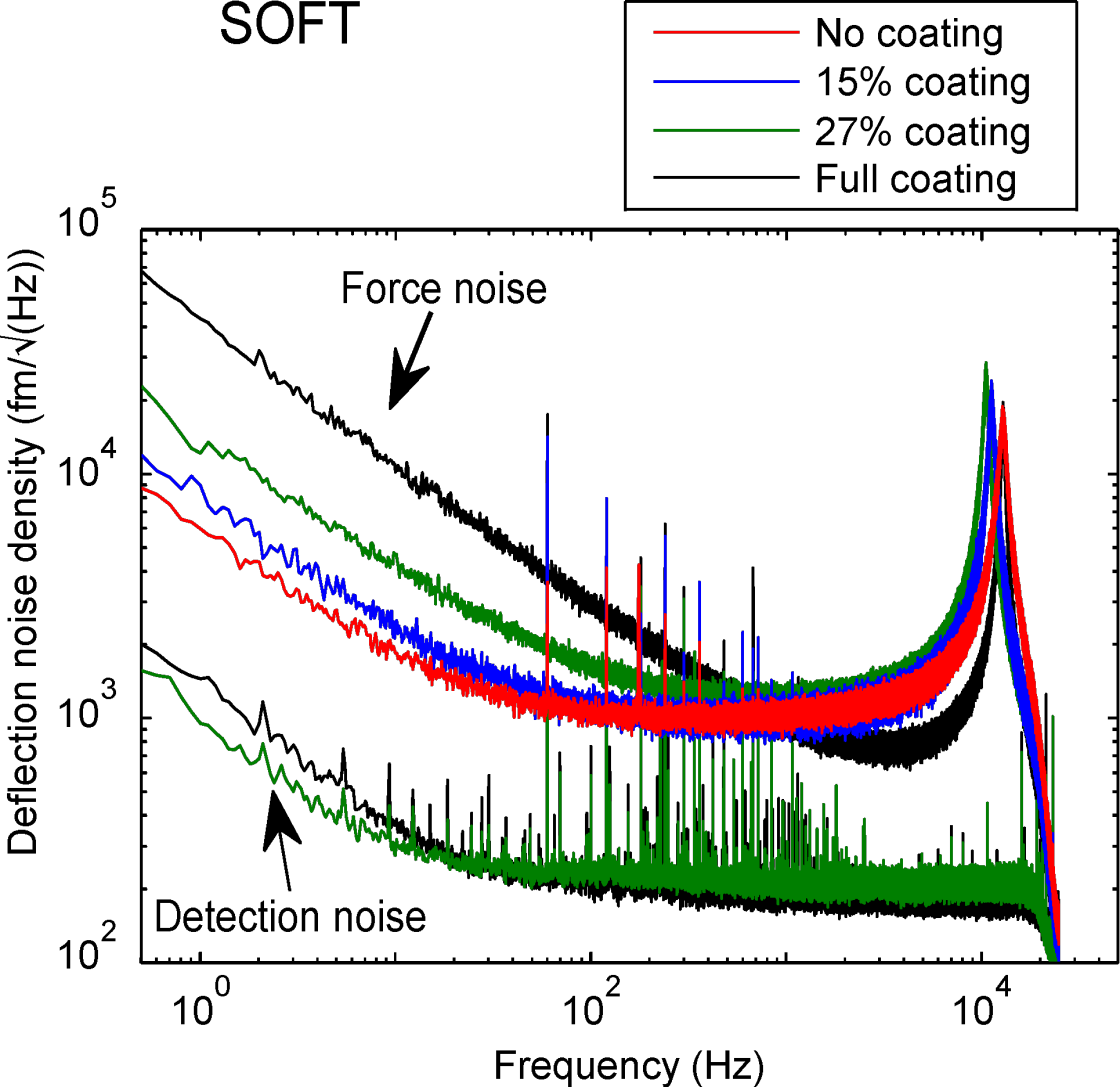
Noise spectra for soft cantilever with different coating coverage acquired in air. Fully coated cantilever shows the highest 1/*f* noise. The 1/*f* noise reduces with reduced coating coverage. The uncoated cantilever shows the lowest 1/*f* noise level.

For static AFM measurement such as contact mode or force spectroscopy, a low 1/*f* noise is important. In this section, the cantilever deflection noise density spectra of the soft cantilevers measured from 1 Hz to 25 kHz in air is discussed. These spectra include the 1/*f* noise as well as the fundamental resonance of the cantilever at 11 kHz. In Figure 2, we compare the noise density spectra of a fully coated, two partially coated and an uncoated cantilevers. A nearly an order of magnitude increase in 1/*f* noise can be observed for the fully coated cantilever compared to the uncoated one. At 1 Hz, the cantilever with a coating coverage of 27% shows a 4 fold reduction in 1/*f* noise compared to the full coating. When reducing the coating coverage even further to 15% only a slightly higher 1/*f* noise compared to no coating is observed.

The two lowest spectra in Figure 2 show the equivalent deflection noise density due to the instrumental noise measured by reflecting the laser beam off the cantilever chip. The measured spectra were converted to the equivalent deflection noise density spectra by the optical beam lever sensitivities obtained for partially and fully coated cantilevers. It is clear that the instrumental noise is much smaller than the observed cantilever noise and the reduction in 1/*f* noise is therefore a true reduction in the force noise (noise due to the cantilever deflection). The reduction is due to the reduced photothermal (bimetallic) effect. Note that the sharp peaks in the spectra are of electronic origin as they also appear in the detection noise. We observed the similar reduction in 1/*f* noise for the NCLR cantilevers, which can be seen in Supporting Information File 1, Figure S2 and Figure S3.

**Figure 3:**
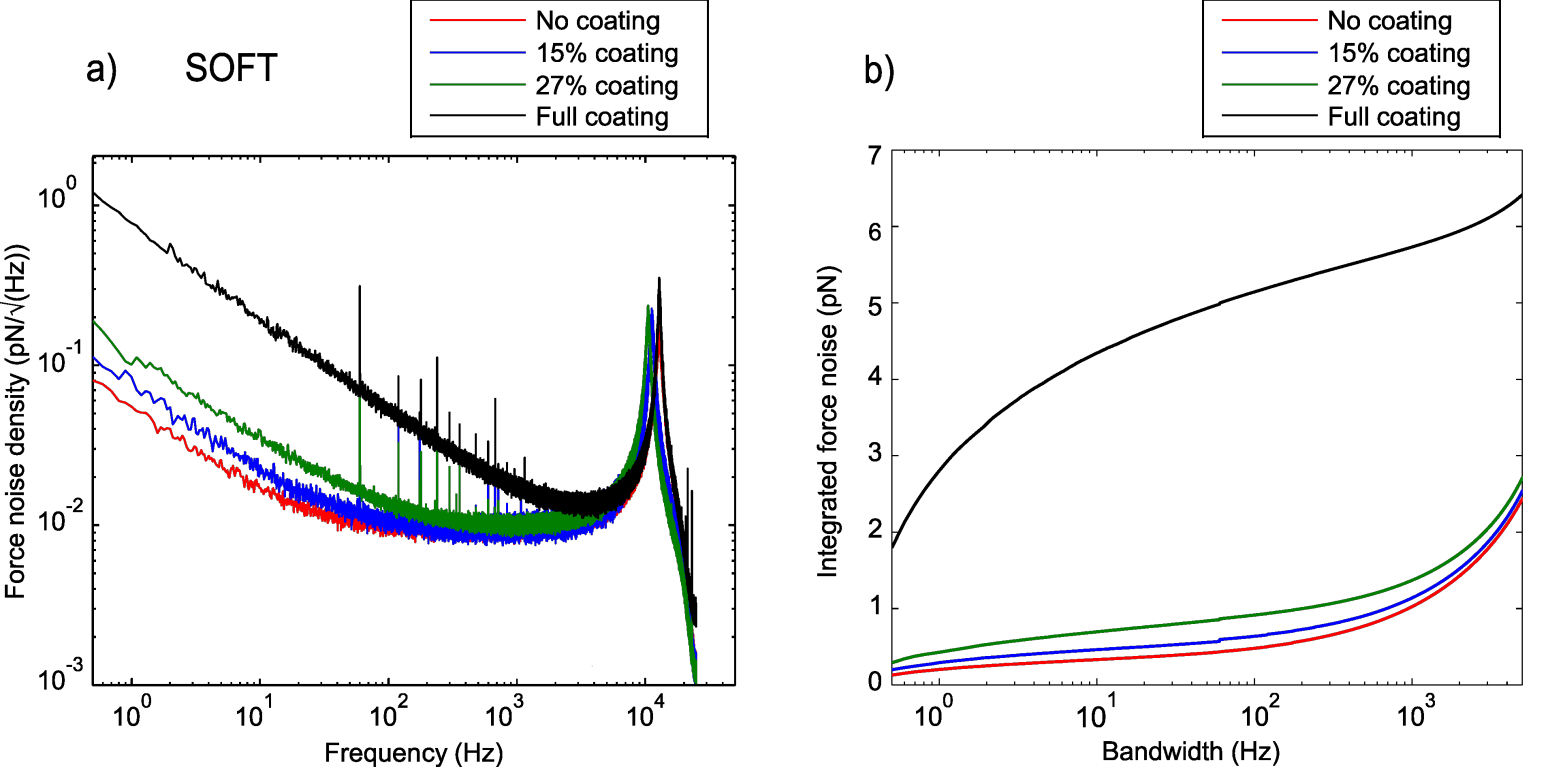
(a) Force noise density spectra for the soft cantilevers obtained from Figure 2 by multiplying with the measured spring constants. (b) The force noise density is integrated to show the expected force noise at a certain bandwidth (0.3 Hz–5 kHz). This sets the minimum force noise with these cantilever, independent of the measured signal. The partially coated cantilever and uncoated cantilever show a sub-pN force noise over a wide range of possible bandwidth (0.5 Hz–1 kHz), whereas the fully coated cantilever shows a strong increase.

In force measurements the force noise is more relevant than the deflection noise itself as it directly shows the performance of the cantilevers as a force sensor (see Figure 3a). The equivalent force noise density spectra were obtained by multiplying the deflection noise density shown in Figure 2 with the measured spring constant of each cantilever. Here, the difference between fully and partially coated cantilevers becomes even more pronounced due to the higher spring constant of the fully coated cantilever.

An additional measure to quantify the noise for force spectroscopy measurements, is the integrated force noise shown in Figure 3b. The integrated force noise shows the expected noise at the corresponding measurement bandwidth, independent of the measured force. It is therefore the minimum force precision achievable by the cantilever in such a static measurement, not to be confused with the minimal detectable force gradient mentioned for FM-AFM in Equation 1. One can clearly see that the noise on the fully coated cantilever increases rapidly in the low frequency range, whereas the partial and uncoated cantilever show a sub-pN force noise up to 1 kHz bandwidth. Bull, et al. [5] previously used the integrated force noise to characterize the noise of cantilevers and verified this parameter experimentally.

**Figure 4:**
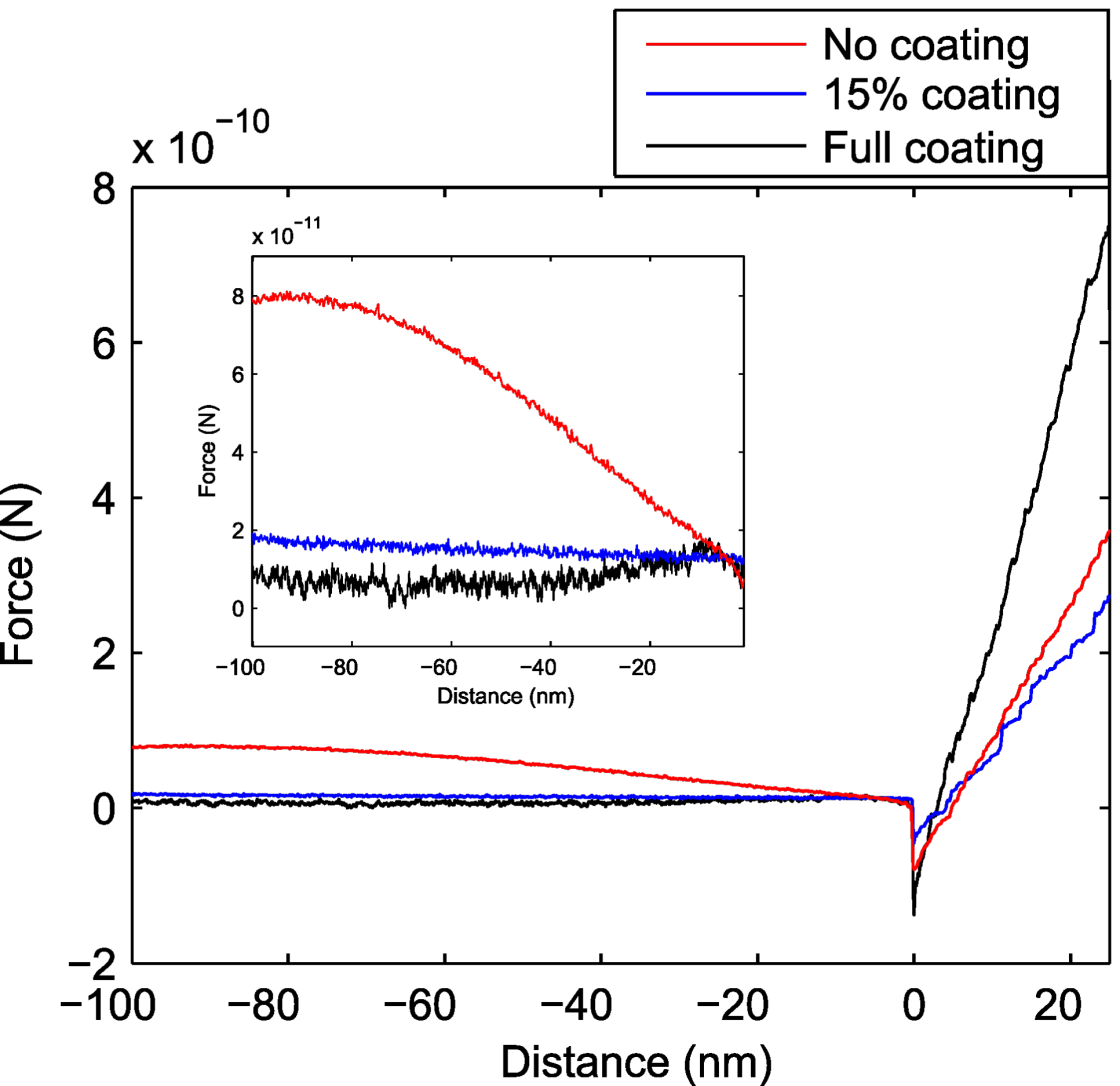
Force–distance curves for an uncoated (red), partial coated (blue) and fully coated soft cantilever (black) with a measurement bandwidth of 1 kHz. In the inset, the approach region is plotted for better illustration of the noise. One can see that the fully coated cantilever shows the highest noise. The uncoated cantilever shows largest variation of the force before contact which is due to the optical interference of the detection laser beam.

Force–distance curves of the soft cantilever on a silicon wafer were taken to show the noise behaviour under more realistic experimental condition. In Figure 4 one can see the increased noise for the fully coated cantilever with an RMS of 2.14 × 10^−9^ N compared to the partially coated and uncoated cantilever (1.80 × 10^−10^ N and 2.01 × 10^−10^ N, respectively). This order of magnitude difference is larger than the systematic error due to the uncertainty in the spring constant. The slowly varying forces appearing before contact are due to optical interference effect of the detection laser beam. The uncoated cantilever shows the largest variation, possibly due to more light being reflected off the sample underneath the cantilever.

### Effect of coating thickness on *Q*-factor

Sosale et al. [8], derived a quantitative theory of how the internal material friction of a partial coating effects the *Q*-factor of a microcantilever:

[2]
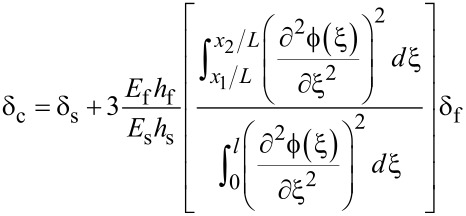


with ξ the normalized length (*l*/*L*), 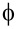
(ξ) the natural mode shape of the cantilever, *E* the Young’s modulus and *h*_f_, *h*_s_ being the coating film thickness and the cantilever thickness. The logarithmic decrement is δ = π/*Q*. The c stands for the composite system, f for the film and s for the substrate. This assumes no clamping losses and a substrate operating at the fundamental thermoelastic limit of dissipation [8].

**Figure 5:**
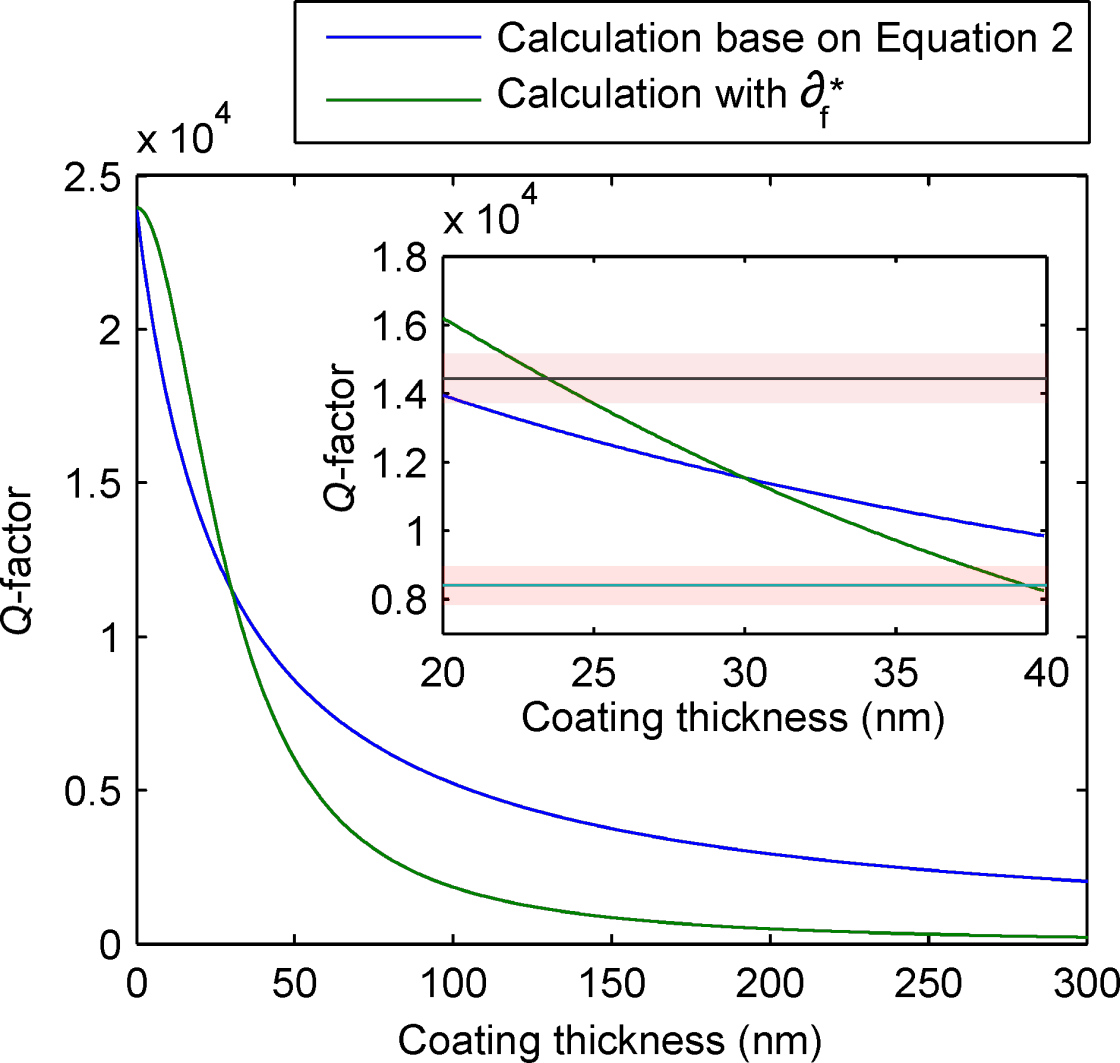
Calculation of the change of *Q*-factor for a fully coated NCLR cantilever with different coating thicknesses. Blue shows the predicted value according to Equation 2. Green shows the predicted *Q*-factor value with the added 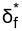 term. The inset shows a zoom to 20–40 nm. The mean measured *Q*-factor for two sets of NCLR with a coating thickness difference of 16 nm are plotted as a horizontal line with standard error by red bands.

We calculate the *Q*-factor dependence on the coating thickness of the NCLR cantilever. Therefore, we measured the *Q*-factor of fully coated and uncoated NCLR cantilevers, to extract the δ_s_ = 4.18 × 10^−5^, δ_f_ = 8.56 × 10^−3^ term in Equation 2. We used these values to plot the *Q*-factor vs coating thickness for coating thicknesses between 0–350 nm on a fully coated NCLR cantilever, see Figure 5.

One should notice that a change in coating thickness of a few nanometer around the standard coating thickness of 30 nm can result in a drastic change in the *Q*-factor, even for a 30 nm coating on a 7 μm thick cantilever. To verify how well this model works for an actual AFM system with clamping losses, the *Q*-factor of two sets of NCLR cantilevers with a difference in coating thickness of 16 nm was used. For each coating thickness at least 18 cantilevers were measured (horizontal line in Figure 5 inset). The average measured difference in *Q*-factor between the two different thicknesses was measured to be 6032 with a standard error of 905, which is larger than that expected from Equation 2.

However, if we assume that the logarithmic decrement of the coating film scales linearly with the thickness of the coating such as 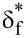 = (δ_f,measured_/*h*_f,measured_)*h*_f_ we replace δ_f_ with 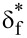 in Equation 2, the modified Equation 2 gives a better agreement with our observation as shown in Figure 5. Nevertheless, one can see in Figure 5 that a small variation in coating thickness for a fully coated cantilever will influence the *Q*-factor significantly for both cases.

## Conclusion

We showed the improved behavior in *Q*-factor and 1/*f* noise for partially over fully/uncoated commercial AFM cantilevers, which is summarized in Table 2. In general thin-film coatings significantly reduce the *Q*-factor of any cantilever, even for coating to cantilever thickness ratios as small as 30 nm/7 μm < 10^−2^ and is therefore relevant for AFM applications. This can be described by the additional viscoelastic damping due the metal coating on the cantilever. The effect of this damping increases with increasing coating to cantilever thickness ratio, which was demonstrated with two types of cantilevers used in this study (soft and NCLR). A larger ratio results in an increased damping, hence in a reduction in *Q*-factor and an increase in 1/*f* noise.

**Table 2:** Summary of the performance of cantilevers with different coating. The partially coated cantilever combines the advantages of the fully coated with the advantage of the partially coated cantilever.

Coating	Signal on diode	*Q*-factor	1/*f* noise

Partially coated	high	high	low
Fully coated	high	low	high
Uncoated	low	high	low

However, the damping due to the coating can be overcome if a partial coating at the tip end of the cantilever is used.

We showed that for soft cantilevers (≈0.01 N/m), a significant reduction in 1/*f* noise can be achieved, which is extremely relevant for static force measurements. For stiffer cantilevers commonly used in FM-AFM, a partial coating with 20% coverage at the tip end of the cantilever retains a similar *Q*-factor as uncoated cantilevers, with the added benefit of a higher signal on the photodiode.

Furthermore, the partial coating of 20% helps to align the laser reliably to the same position on the cantilever since the intensity of the reflective signal decrease significantly when the beam is moved in any of the four direction away from the coating. This should help to achieve more reproducible deflection sensitivity measurement since they depend on the position of the laser beam on the cantilever [13].

We also showed that a slight variation in coating thickness can result in significant changes in the *Q*-factor of a cantilever. Therefore, fabrication dependent variations of the coating thickness will influence the *Q*-factor. If a partial coating is used, this effect becomes unimportant, resulting in more reproducible Q-factors from fabrication batch to batch.

In summary, there is no need for fully coated cantilevers since the coating reduces the *Q*-factor in UHV and adds 1/*f* noise for soft cantilever. The coating at the base of the cantilever is not needed since the sole purpose of the coating is to reflect the laser beam at tip end of the cantilever. Partially coated cantilevers would therefore be a better choice for a variety of AFM applications.

## Supporting Information

The Supporting Information includes *Q*-factor measurments for the soft cantilver and deflection noise density spectra for the NCLR cantilever.

File 1 Detection noise measurement for NCLR cantilever.
