# Supplementary material for: Improved atomic force microscopy cantilever performance by partial reflective coating
Source: Beilstein J Nanotechnol. 2015 Jul 3;6:1450–6. doi: 10.3762/bjnano.6.150 (PMC4505110; doi:10.3762/bjnano.6.150)
Supplement: File 1 — Detection noise measurement for NCLR cantilever. [file Beilstein_J_Nanotechnol-06-1450-s001.pdf]

Supporting Information

for

Improved atomic force microscopy cantilever performance by partial reflective coating

Zeno Schumacher*[1], Yoichi Miyaharaa[1], Laure Aeschimann[2] and Peter Grütter[1]

Address: [1]Department of Physics, McGill University, Montreal, Quebec, H3A 2T8, Canada and [2]NanoWorld AG, Neuchâtel, 2002, Switzerland

Email: Zeno Schumacher - zenos@physics.mcgill.ca

* Corresponding author

Detection noise measurement for NCLR cantilever

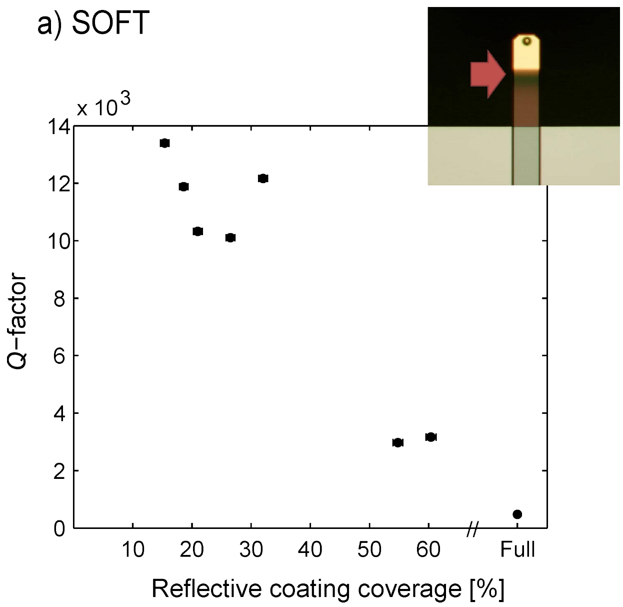

Figure S1: Q-factor of cantilevers with different amount of coating coverage. a) The soft cantilever is 340 nm thick with a 60 nm thick Au coating. The partial coating is located at the tip end of the cantilever (see inset). 15 % coating show a 28 times increase in Q- factor compared to the fully coated cantilever.

Figure S1 shows the Q-factor of the soft cantilever measured under high vacuum condition from the excited resonance spectra. The highest increase was observed with a coating coverage of 15%, resulting in a 28 times increase in Q-factor. An average increase of 24 times for partial coatings that cover less than 35% of the cantilever was observed. This shows that the increase in Q-factor with reduced coating can be seen for soft cantilever under vacuum. However, these cantilever are not commonly used under vacuum condition or for FM-AFM.

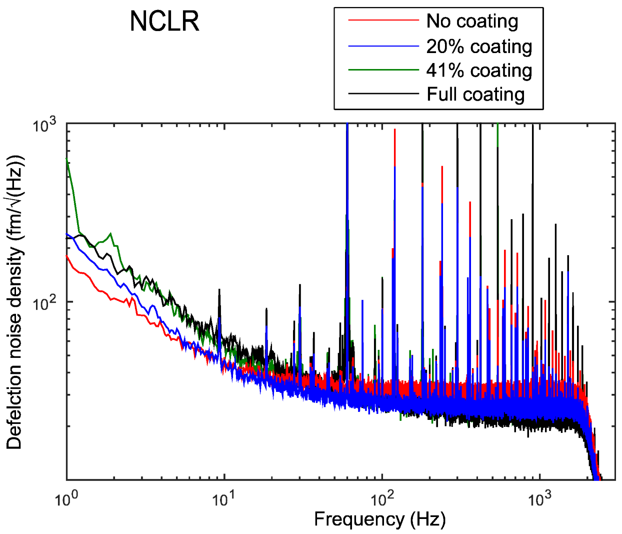

Figure S2: Low frequency noise spectrum for NCLR cantilevers measured in high vacuum $< 5 \times 10^{-5}$ mBar. A decrease in $1/f$ noise is measurable, however not as strong as for the soft cantilevers due to the different coating and cantilever thickness.

Figure S2 shows the deflection noise spectra density for the NCLR cantilever measured between 1 Hz to 2 kHz in high vacuum condition. A 1.6 fold reduction in $1/f$ noise as a result of a smaller coating coverage is observed. However, the reduction is not as much as that for the soft cantilever. We attribute the smaller reduction to the coating to cantilever thickness ratio (h_f/h_s, see Table 1), which is much smaller for the NCLR cantilever.

The uncoated NCLR cantilever shows a higher noise level around 1 kHz compared to the partially or fully coated ones. This is due to the decreased optical lever sensitivity of the uncoated cantilever. This measurement clearly demonstrates how the partially coated cantilever combines the advantages of the uncoated and fully coated cantilever. Partial coating leads to a decrease in $1/f$ noise compared to fully coated cantilever, as well as less noise at higher frequencies (above 100 Hz) compared to the uncoated cantilever. These leads to an advantage over both the fully coated and uncoated cantilever.

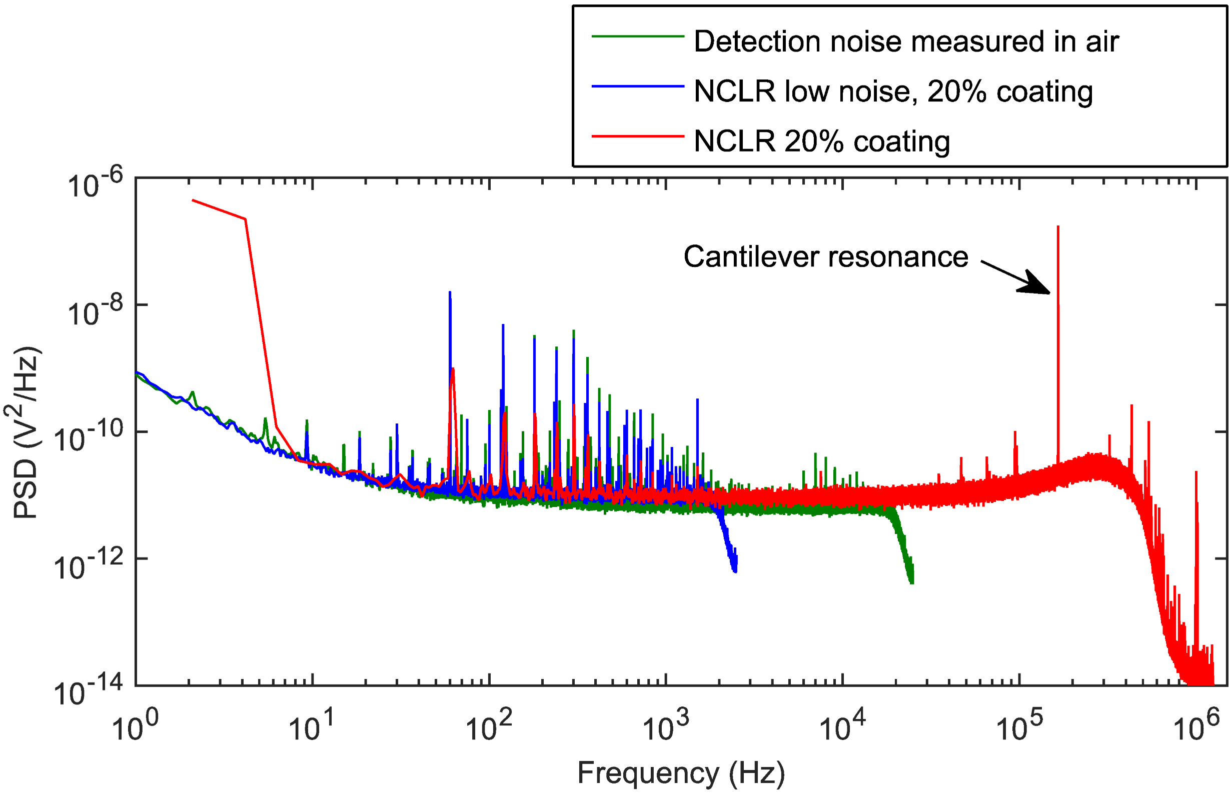

Figure S3: Raw data for detection noise (green), and partial coated NCLR cantilever (red and blue). The measured noise for the NCLR cantilever is limited by the detection noise of our system. The peak appearing between 10 Hz–1 kHz are electronic noise peaks.

Overall the $1/f$ noise is much lower for the NCLR type than for the soft cantilever. One should note as well that peaks appearing between 100 Hz–1 kHz. These peaks originate from the detection system, which can be seen in Figure S3. A comparison of the raw data reveals that the noise measurement for the NCLR cantilever is actually limited by the detection noise. Therefore the electronic peaks present in the detection noise appear in the force noise measurement as apparent force noise peaks, even though their origin is in the electronic detection system. Since the noise measured for our NCLR cantilever lies just above the detection noise, it is possible that the biggest contribution to the entire noise comes from the detection noise rather than from the cantilever itself.
